# Precision Cancer Medicine 2.0—Oncology in the postgenomic era

**DOI:** 10.1002/1878-0261.13707

**Published:** 2024-08-07

**Authors:** Riccardo Masina, Carlos Caldas

**Affiliations:** ^1^ Cancer Research UK Cambridge Institute and Department of Oncology, Li Ka Shing Centre University of Cambridge UK; ^2^ School of Clinical Medicine University of Cambridge UK

**Keywords:** cancer, data integration, machine learning, precision cancer medicine, translational research, tumour biomarkers

## Abstract

Genomic medicine has transformed the lives of patients with cancer by enabling individualised and evidence‐based clinical decision‐making. Despite this progress, the implementation of precision cancer medicine is limited by its dependence on isolated biomarkers. The development of bulk and single‐cell multiomic technologies has revealed the enormous complexity of the cancer ecosystem. Beyond the cancer cell, the tumour microenvironment, macroenvironment and host factors, including the microbiome, profoundly influence the cancer phenotype, and accounting for these enhances the resolution of precision medicine. The advent of robust multiomic profiling and interpretable machine learning algorithms mark the dawn of a new postgenomic era of personalised cancer medicine. In Precision Cancer Medicine 2.0, high‐resolution personalised clinical decision‐making is informed by the comprehensive multiomic profiling of tumour and host, integrated using artificial intelligence.

## Graphical abstract

The implementation of precision cancer medicine today is limited by its dependence on discrete biomarkers. The advent of robust multiomic profiling and interpretable machine learning algorithms marks the dawn of a new postgenomic era of personalised cancer medicine. In Precision Cancer Medicine 2.0, personalised clinical decision‐making is informed by the comprehensive multiomic profiling of tumour and host, integrated using artificial intelligence.

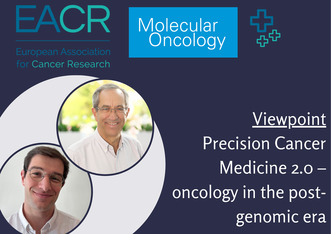

**Abbreviations**
AI: artificial intelligence
CCS: circulating cancer secretome
ctDNA: circulating tumour DNA
ECM: extracellular matrix
HRD: homologous recombination repair deficiency
PCM2.0: Precision Cancer Medicine 2.0
TME: tumour microenvironment

## 1 Precision Cancer Medicine 2.0

For the past 50 years, biology has been the engine of exceptional progress in our understanding of cancer. The use of elegant model systems has produced extraordinary insights into the inner workings of tumours that converged in the Hallmarks of Cancer [1].

**Fig. 1:**
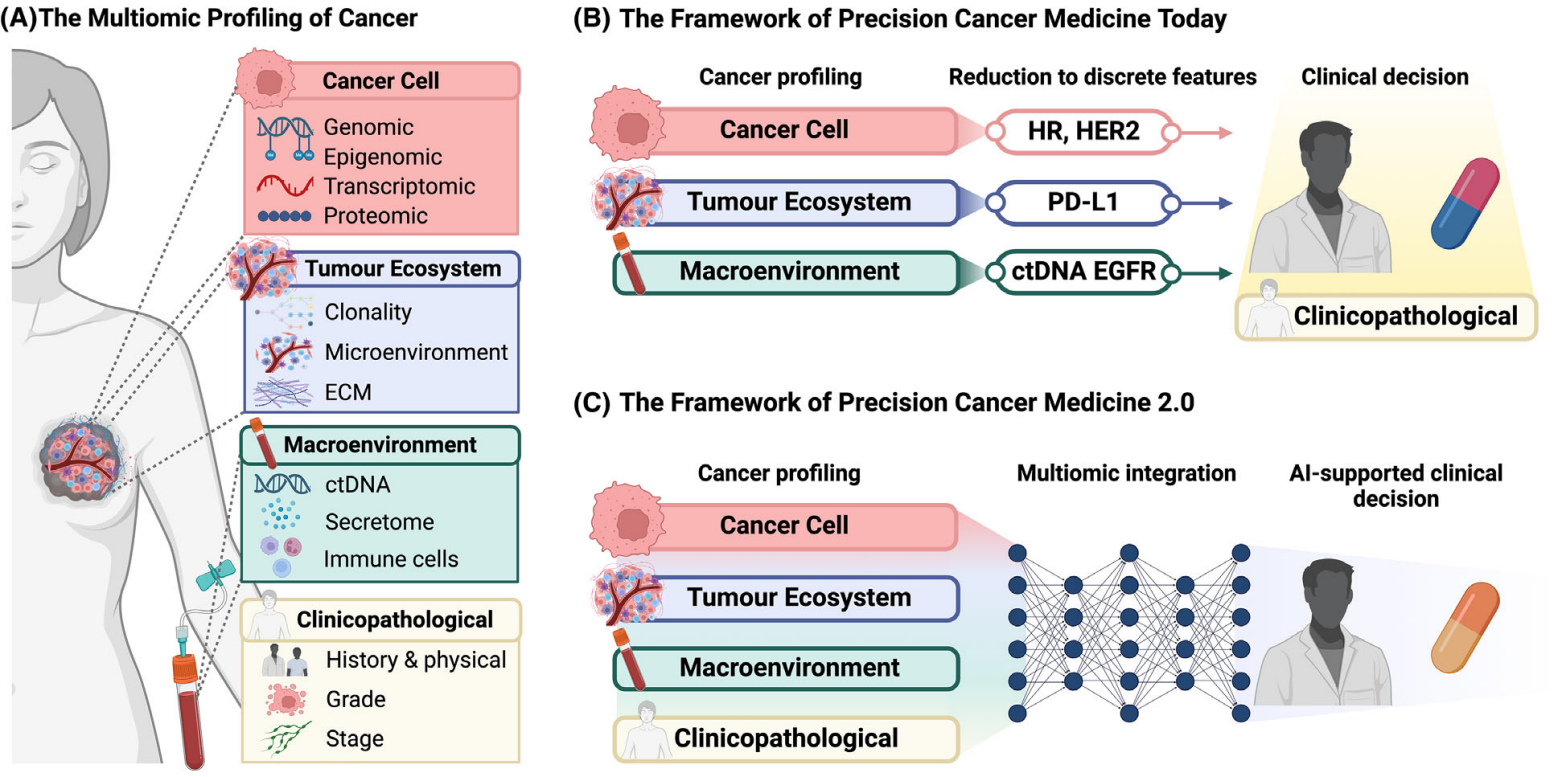
(A) Schematic of the sources of data for comprehensive multiomic profiling of cancer patients. (B) Framework of Precision Cancer Medicine today, in which clinical decision‐making is informed by discrete features. (C) Framework of Precision Cancer Medicine 2.0, in which the entire multiomic profiles of tumour and host are integrated using artificial intelligence algorithms to achieve high‐resolution personalised clinical decision‐making. AI, artificial intelligence; ctDNA, circulating tumour DNA; ECM, extracellular matrix; HR, hormone receptor.

This strategy has led to the identification of actionable molecular biomarkers to tailor anticancer treatment, opening the doors to genomic medicine: individualised and evidence‐based clinical practice, which has transformed the lives of cancer patients. Namely, the overall 5‐year survival rate in 2017 was 70%, whereas in the 1950s, it was only 37% [1, 2]. Although the complexity of cancer is well‐acknowledged, the implementation of precision cancer medicine until now has relied on discrete sets of isolated biomarkers (e.g. HR, HER2 and BRCA1/2 in breast cancer) [2]. This strategy is inherently restricted, because in fact, the same cancer phenotype can arise from countless combinations of different alterations. Oncology must capture the order that exists beyond the diversity of individual alterations. As multiomic profiling becomes robust and affordable, and machine learning algorithms become widely interpretable and efficient, we are at the dawn of a new postgenomic era of personalised cancer medicine. In Precision Cancer Medicine 2.0 (PCM2.0), the comprehensive profiles of tumours and hosts are fed whole into artificial intelligence (AI) algorithms to achieve truly personalised clinical decision‐making. This viewpoint outlines how disparate data must be integrated to deliver PCM2.0, encompassing the cancer cell, the tumour ecosystem, the tumour macroenvironment and host factors, including the microbiome (Fig. 1).

## 2 The cancer cell

The implementation of personalised cancer medicine has centred on the identification of actionable biomarkers within the cancer genome. The discovery of oncogenes and tumour suppressor genes has allowed the development of tools for screening, diagnosis, prognosis, prediction and therapeutic targeting [2]. However, the translation of this knowledge into clinical practice has faced two limitations that must be overcome to deliver PCM2.0.

The clinical utility of any isolated biomarker is hindered by the diversity of the inner workings of tumours. To tackle this, efforts are in place to condense the genomic diversity into biologically and clinically informative pathways across molecular layers at the whole‐genome scale [3]. For instance, the discovery of genomic signatures that capture the endogenous and exogenous sources of cancer mutagenesis such as Homologous Recombination Repair Deficiency (HRD) enables clinical decision‐making beyond the granularity permitted by isolated driver mutations [4, 5]. Soon, decisions to treat with PARP‐inhibitors could include HRD status alongside traditional BRCA_WT/MUT_ testing [5].

The second barrier to the translation of cancer genomics is the gap that still exists between genomic data and a true understanding of cancer biology. The integration of multiomic profiles of tumours beyond genomics, to encompass transcriptomics, epigenomics and proteomics, is bridging this gap to yield novel mechanistic insights that enable rational understanding of the behaviour of cancer cells.

## 3 The tumour ecosystem

The development of multiomic technologies at single‐cell resolution has revealed that tumours are dynamic heterogeneous mixtures of malignant clones that evolve under Darwinian pressures from both therapy and immune response. This exposes a major challenge to effectively curing cancer patients, as heterogeneous mixtures of cancer cells and the tumour microenvironment (TME) respond differently to the same selective pressures. Precision medicine tools that account for intratumoral heterogeneity outperform those that rely solely on bulk profiles [6, 7].

The use of TME‐based biomarkers is already part of clinical practice. Quantification of PD‐1 and tumour‐infiltrating lymphocytes informs treatment decisions with immune checkpoint blockade. Again, these are used as isolated features, sacrificing biomarker specificity for practicability. Spatial technologies such as spatial transcriptomics, multiplex high‐resolution imaging and niche‐labelling strategies have shown that the multicellular organisation of the TME and extracellular matrix (ECM) contribute critically to the cancer phenotype. Machine learning models that account for the spatial architecture of tumours in 2D, and increasingly in 3D, are able to reliably predict clinical outcomes such as prognosis and response to immune‐checkpoint inhibition [6, 8].

This enormous cancer‐intrinsic complexity and heterogeneity leads to a massive combinatorial problem when testing existing and novel therapies. More predictive preclinical models and imaginative novel clinical trial designs are urgently needed to deliver on the promise of PCM 2.0.

## 4 The tumour macroenvironment

The perturbations mediated by carcinogenesis extend beyond the local microenvironment to induce profound alterations in the patients' immune, metabolic, cardiovascular and neuro‐endocrine systems. These can be assayed in blood, and great effort is spent on the systematic profiling of its components, including circulating tumour DNA (ctDNA), cancer secretome and immune cells.

### 4.1 Circulating tumour DNA

It has become clear that ctDNA can be detected in the plasma of cancer patients and correlates with the cancer genotype and epigenotype. ctDNA characterisation initially relied on quantitative and digital PCR assays. It is now predominantly performed using panels or whole‐genome sequencing yielding comprehensive genome‐wide profiles that can be integrated using AI to augment diagnosis, monitoring and treatment. Furthermore, liquid biopsies, often performed alongside tissue biopsies, allow noninvasive serial tracking of tumour clonal dynamics and evolution [2, 9].

### 4.2 Cancer secretome

Mass spectrometry‐based assays have enabled the interrogation of thousands of proteins and metabolites including lipids in bulk tissues and at single‐cell resolution. This technology also allows the characterisation of the circulating cancer secretome (CCS), which reflects processes such as metastatic disease, cancer‐associated cachexia, venous‐thromboembolism, cardiovascular disease, neuro‐endocrine dysfunction and immunosuppression. Information from the CCS enhances the accuracy of clinical predictions [10].

### 4.3 Immunophenotyping

The systemic immune status of oncological patients is intricately linked to the cancer phenotype. The analysis of the peripheral blood immune compartment of patients has clinical utility [11]. Multiparameter flow cytometry, T‐cell receptor and B‐cell receptor sequencing, antibody profiling and single‐cell multiomics allow immunophenotyping at unprecedented resolution and inform machine learning algorithms that can reliably predict clinical outcomes such as response to immunotherapy [11, 12]. A comprehensive understanding of the tumour circulating compartment, including ctDNA, plasma/serum secretome and immune cells, increases the resolution of precision medicine.

## 5 Clinical characteristics

Importantly, accurate and robust clinical decision‐making can already be achieved today by relying solely on known clinicopathological factors that retain independent significance on multivariate analysis such as tumour size, tumour grade, lymph node status, previous treatment and demographic characteristics such as age, sex and past medical history [13, 14, 15].

The implementation of PCM2.0 cannot ignore the information obtained from conventional clinical practice, including radiology and functional imaging. The inclusion of multiplexed profiling of tumour, TME, macroenvironment and host will augment but it will not replace this information, increasing the resolution of clinical predictions. Electronic health records, with appropriate meta‐data tags, will be enormous facilitators of PCM2.0.

## 6 Artificial intelligence to deliver PCM2.0

The foundation for the next generation of personalised medicine relies on the unprecedented availability of comprehensive multiomic profiling data. Deeply phenotyped cancer data sets are complex and require AI to transform information into actionable knowledge. Our recent publication has reported a framework that marks the dawn of PCM2.0 [14].

Data availability remains a bottleneck for the widespread application of PCM2.0. Comprehensive profiling of cancer requires resources and expertise, which poses a limit to the number of profiles that can be generated by any provider. Equally, the more comprehensive the profiling, the larger the sample size required for AI training. This trade‐off between sample size and throughput is so problematic that the accuracies of most published integrative AI algorithms today are not saturated, meaning they will improve further when larger data sets become available for additional model training [14, 15].

Equity of access poses another bottleneck to the implementation of PCM2.0. The incorporation of multiomic profiling into the standard of care will lead to disparities between centres with different resources. Strategies are needed to extend access to PCM2.0 beyond the minority of centres that are able to offer multiomic cancer profiling. A technical expedient to mitigate this issue is to ensure that trained integrative AIs are modular—that is that they do not require the entirety of the profiles to output reliable predictions. Another expedient is to harness the power of transfer learning in AI. By training the modules not only to predict the outcome but also to predict each other, one can increase the efficiency of learning from any single‐module data set with knowledge learned from other modules in external large multiomic data sets. Pioneering work has shown that similarly trained AI algoritms can accurately predict tumour types from H&E slides by inferring the methylation profile of a subset of highly informative CpG sites or gene expression, thus bypassing the need for genomic assays [15]. Technical advancements aside, PCM2.0 demands an urgent and well‐supported dialogue among political, academic and industry stakeholders to safeguard global equity of access. Collaborative efforts are essential to overcoming access barriers and disparities.

## 7 Conclusion

Precision Cancer Medicine 2.0 heralds a new era of cancer management and treatment, in which high‐resolution personalised clinical decision‐making is informed by the multiomic profiling of tumour and host, leveraged by interpretable AI. Realising this potential requires addressing challenges in data availability and safeguarding of equitable access. Cancer patients will be the main beneficiaries.

## Conflict of interest

The authors declare no conflict of interest.

## Author contributions

CC first proposed the concept of PCM 2.0. RM and CC conceived and designed this Viewpoint and wrote the paper.
